# Laparoendoscopic Single-Site (LESS) Retroperitoneal Radical Nephrectomy in a Patient with Renal Cell Carcinoma Receiving Hemodialysis

**DOI:** 10.1155/2011/506032

**Published:** 2011-06-02

**Authors:** Takeo Nomura, Fuminori Sato, Mika Takahashi, Yasuhiro Sumino, Hiromitsu Mimata

**Affiliations:** Department of Urology, Faculty of Medicine, Oita University, 1-1 Idaigaoka, Hasama-machi, Yufu, Oita 879-5593, Japan

## Abstract

We present here the patient undergoing laparoendoscopic single-site (LESS) retroperitoneal radical nephrectomy while receiving hemodialysis. An 81-year-old man under hemodialysis for 6 years was incidentally discovered to have two left renal masses with acquired cystic disease of the kidney (ACDK). A 4-cm flank incision for GelPort was made. Three trocars were inserted into the retroperitoneum through GelPort. After division of the renal vessels and ureter, the kidney was placed into the extraction bag and was retrieved through flank incision without any extra skin incision. There were no intraoperative and postoperative complications. This procedure offers an effective, minimally invasive therapeutic alternative to the standard laparoscopic technique in high-risk end-stage renal disease patients.

## 1. Introduction

The incidence of developing renal cell carcinoma (RCC) has been estimated to be more than 20 times higher in dialysis patients compared to the general population [1]. In patients with end-stage renal disease, radical nephrectomy emerges as the standard treatment for localized RCC [2]. Generally, these patients are at increased surgical risk because end-stage renal disease is associated with bleeding tendency, metabolic acidosis, congestive heart failure, and increased susceptibility to infection and as such may require definitive surgical management [3–5]. Therefore, the safe and minimally invasive treatment is desirable in these high-risk patients. 

Laparoscopic radical nephrectomy (LRN) is the most common operation described as minimally invasive surgery with a high success rate and low incidence of complications in RCC patients [6]. Shoma et al. reported a high success rate, low morbidity, and rapid recovery in 62 laparoscopic pretransplant native nephrectomies in patients with end-stage renal disease [7]. In addition, Bird et al. showed that LRN is feasible and safe with acceptable complication rates for the treatment of RCC in patients requiring dialysis [8]. To date, LRN is being increasingly performed and has steadily assumed a central role in managing RCC in patients with end-stage renal disease. Conventional LRN typically requires three to four ports for a given procedure. As a result of the risks associated with additional ports, there has been a surge of interest in a less invasive alternative. A new alternative to conventional laparoscopic surgery is single-port or single-incision laparoscopic surgery. Furthermore, embryonic natural orifice translumenal endoscopic surgery (E-NOTES) via vagina, mouth/stomach, rectum, and umbilicus is a recent innovation [9, 10]. Since Raman et al. performed the first laparoendoscopic single-site (LESS) nephrectomy in 2007 [11], subsequent work has expanded indications to a variety of urological surgeries including pyeloplasty, partial nephrectomy, adrenalectomy, prostatectomy, and donor nephrectomy [12–14]. LESS nephrectomy offers some advantages over conventional LRN including decreased pain, quicker convalescence, and improved cosmesis [15]. We report on the initial case of LESS retroperitoneal radical nephrectomy in a patient receiving hemodialysis. 

## 2. Case Report

**Figure 1:**
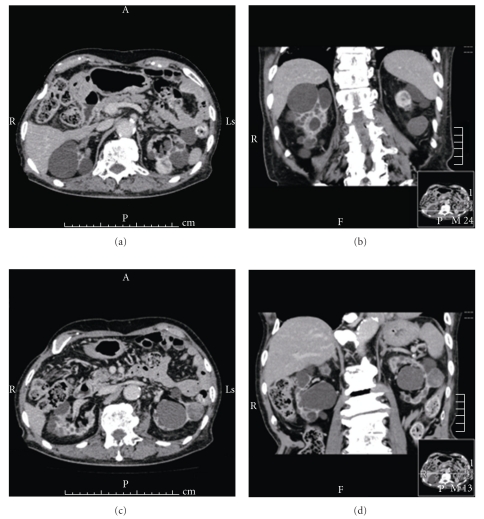
Abdominal computed tomography demonstrated two masses, 2.1 × 2.0 cm and 2.4 × 2.2 cm in diameter, at the middle of the left kidney with acquired cystic disease of the kidney.

**Figure 2:**
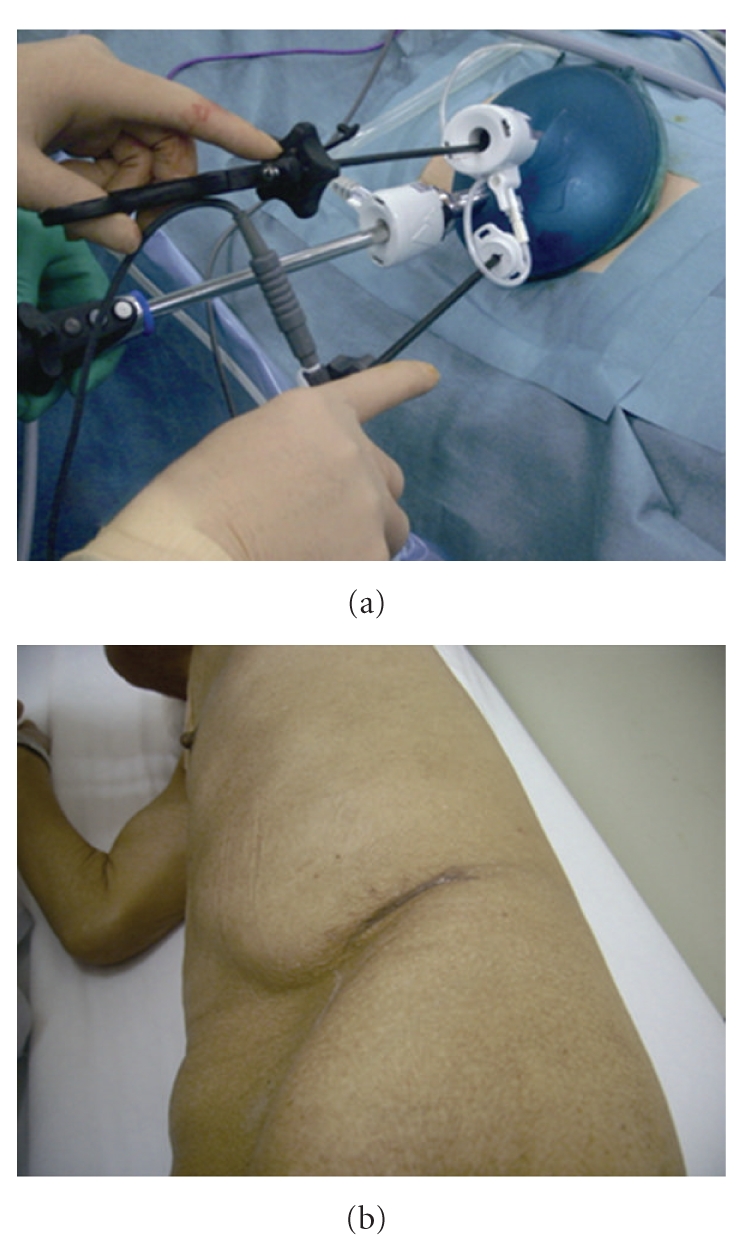
(a) Actual port placement for a left nephrectomy. (b) Photograph of a patient's abdomen 1 month postoperatively.

**Figure 3:**
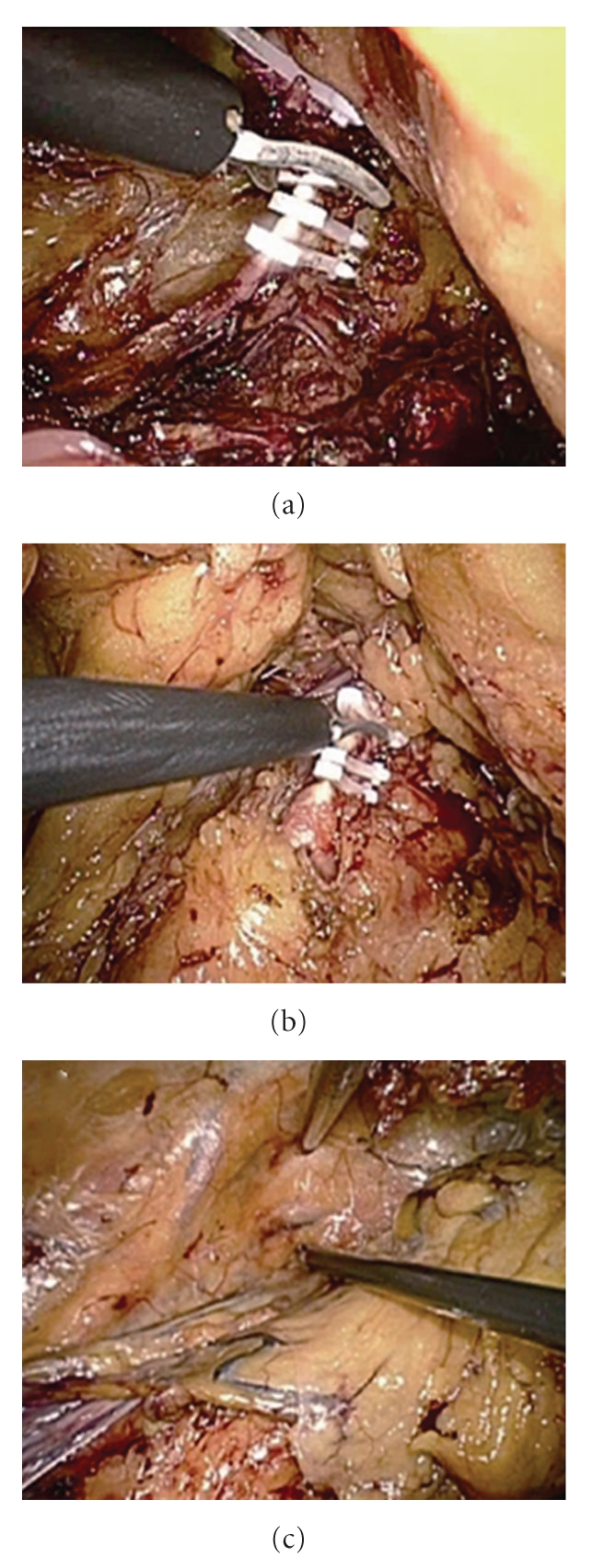
Intraoperative view after renal hilus dissection. (a) Renal artery was divided with Hem-O-Lok clips. (b) The renal vein was clipped with Hem-O-Lok clips. (c) Dissection of the inferior pole of the kidney from the undersurface of the peritoneum in a satisfactory degree of triangulation.

**Figure 4:**
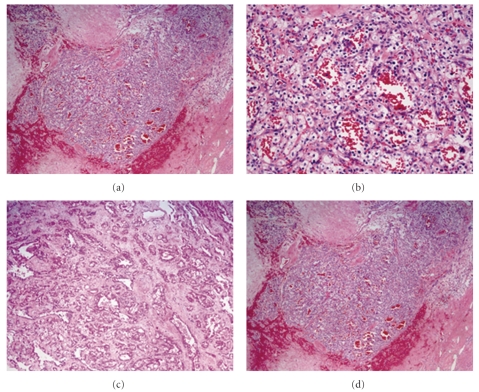
Histological findings (H&E staining) (a) The posterior tumor consisted of clear renal cell carcinoma in alveolar pattern (×40). (b) The tumor cells were round or polygonal with abundant cytoplasm (×200). (c) The lateral tumor was composed solely of eosinophilic cells in tubulocystic pattern (×40). (d) The tumor cells had small nuclei with granular cytoplasm (×200).

An 81-year-old man under hemodialysis for 6 years was incidentally discovered to have two left renal masses with ACDK (Figures 1(a)–1(d)). Following the induction of general anesthesia, the patient was placed in a 80-degree modified flank position with the operating table minimally flexed. A 4-cm flank incision was made in the lumbar region and digital dissection was performed along the anterior surface of the psoas muscle and fascia, posterior to Gerota's fascia. GelPort was placed in the incision as the access platform, which can help to provide adequate spacing, triangulation, and flexibility of port placement. One 10-mm and a 5-mm trocars were placed on either side of camera port on the edges of the GelPort (Figure 2(a)). A 10-mm 30-degree rigid endoscope was used. The posterior surface of Gerota's fascia was sharply opened close to the psoas muscle to expose the fatty tissue harboring renal vessels. The renal artery was dividedand triple-clippedwith Hem-O-Lok clips (size L), and the renal vein was divided with 4 Hem-O-Lok clips (size XL) (Figures 3(a) and 3(b)). After dividing the renal vessels, the inferior pole of the kidney was mobilized from the undersurface of the peritoneum. With gentle caudal traction on the kidney, the upper pole of the kidney was mobilized from the undersurface of the peritoneum and diaphragm. The remaining posterior renal attachments were divided with sharp and blunt dissection, and the kidney was circumferentially mobilized. After lateral attachments to the ureter and kidney were completely incised, the ureter and gonadal vein were clipped and divided. Care was taken to avoid rupturing multiple cysts. Although the kidney was atrophic, dense adhesions at the upper pole of the kidney to the surrounding tissue occurred, and concomitant adrenalectomy was performed due to oozing of blood from the adrenal gland. The kidney was placed into the extraction bag and was retrieved through flank incision without any extra skin incision. The fascia was closed with interrupted sutures, and the skin incision was closed with buried suture after placing a drain tube. Surgical time was 234 minutes, and blood loss was 30 mL. There were no intraoperative and postoperative complications. The patient started the oral intake on the evening of the surgery, and ambulation began on the first postoperative day. The resected specimen was pathologically diagnosed as clear cell carcinoma and renal oncocytoma with ACDK (Figures 4(a)–4(d)). The skin incision was a small single scar 1 month postoperatively (Figure 2(b)).

## 3. Discussion

Patients with end-stage renal disease have increased the risk of developing RCC with an estimated incidence of more than 20 times higher compared to the general population [1]. ACDK would develop in 40–50% of patients receiving hemodialysis, and the development of ACDK is dialysis duration-dependent process and is not influenced by the dialysis mode either in hemodialysis or peritoneal dialysis [16]. Furthermore, a higher incidence of RCC, either multiple or bilateral, has been reported in dialysis patients with ACDK [17]. Radical nephrectomy should be the standard treatment in dialysis patients even for small tumors because nephron-sparing surgery is not beneficial in patients with end-stage renal disease. In fact, satellite tumors are present in approximately 30% of the patients [2]. In addition, it does not seem reasonable to jeopardize the cancer control in an attempt to spare nephrons almost nonfunctioning. Generally, these patients are considered high-risk operative candidates because end-stage renal disease is associated with bleeding tendency, metabolic acidosis, electrolyte imbalances, anemia, hypertension, congestive heart failure, and increased susceptibility to infection [3–5]. In addition, the anticoagulants utilized for hemodialysis may enhance bleeding during operation and postoperative period. Therefore, the safe and minimally invasive treatment is desirable in these high-risk patients. 

Since Clayman's initial description of LRN in 1991 [18], this procedure has rapidly gained worldwide acceptance as minimally invasive surgery with a high-success rate and low incidence of complications in RCC patients [6]. Bird et al. showed that LRN is feasible and safe with acceptable complication rates for the treatment of RCC in patients requiring dialysis [8]. To date, LRN is being increasingly performed and has steadily assumed a central role in managing RCC in patients with end-stage renal disease. Although LRN has many advantages over conventional open nephrectomy, including far less injury, decreased pain, quicker resumption of oral intake, a short hospital stay, cosmesis, and few complications, it still requires several incisions, each at least 1-2 cm in length. Each incision carries potential morbidity risks of bleeding, pain, and hernia and may decrease cosmesis. With the aim of preventing port-site complications, decreasing discomfort associated with laparoscopic surgery, and improving cosmesis, NOTES and LESS have recently been developed. Since Raman et al. performed the first LESS nephrectomy in 2007 [11], subsequent work has expanded the indications to a variety of urological surgeries including pyeloplasty, partial nephrectomy, adrenalectomy, prostatectomy, and donor nephrectomy [12–14]. 

LESS nephrectomy offers some advantages over conventional LRN including decreased pain, quicker convalescence, and improved cosmesis [15]. The main issue of LESS nephrectomy is the need for significant prior laparoscopic experience and the steep learning curve related with this procedure. There are certain technical issues that need to be competitive with conventional laparoscopy. First, unlike conventional laparoscopy, placing several parallel instruments makes triangulation more difficult. However, with the use of GelPort, the instruments can be arranged at 3-cm intervals and a satisfactory degree of triangulation is accomplished. In this case, we achieved good triangulation with standard laparoscopic instruments in the retroperitoneal space (Figure 3(c)). We suggest that articulating or bent instruments will be suitable for transperitoneal approach but not for retroperitoneal access because the distance to target organ from the skin incision is shorter in the retroperitoneal space. Second, close coordination is required between the surgeon and the scopist due to the parallel placement and close proximity of the instruments. In fact, the laparoscope tends to clash with working instruments and/or the operator's hand easily comes in contact with scopist's hand. To avoid this significant discomfort, we preferred to use instruments of different lengths. Third, the lack of additional trocars makes retraction more difficult. It is hard to expose the structures correctly by using single working instrument. In this case, we completed operation without additional trocars, but a satisfactory retraction can be achieved by transcutaneous sutures grasped with extracorporeal handling. It should be emphasized that whenever we have a risk of injuring the patient, additional trocars should be placed and converted to standard laparoscopy without hesitation. 

In our opinion, LESS retroperitoneal radical nephrectomy has surgical challenge. Although LESS transperitoneal approach seems to be easier in a wide working space and many localizing landmarks, the benefits of retroperitoneal approach are little possibility of intra-abdominal organ injury and ligation of renal vessels prior to dissection of the kidney, in particular, for patients with a history of previous abdominal surgery and adhesions. Indeed, the major disadvantage of retroperitoneal approach is a narrow working space that may render it unsuitable for nephrectomy of large kidneys, but the kidneys in patients with dialysis are typically small and atrophic and are thus well suited for removal by LESS. In addition, preservation of the peritoneum is important for patients with hemodialysis in view of possible conversion to peritoneal dialysis. The main disadvantage is the longer operative time, which decreased as we gained more experience. The safe and successful development of LESS has the potential to become a standard for the treatment of small renal tumors in atrophic kidneys secondary to chronic renal failure in the continuing evolution of minimally invasive surgery.
